# Supplementary material for: A sex-specific evolutionary interaction between ADCY9 and CETP
Source: eLife. 2021 Oct 5;10:e69198. doi: 10.7554/eLife.69198 (PMC8594919; doi:10.7554/eLife.69198)
Supplement: Transparent reporting form [file elife-69198-transrepform1.pdf]

Westbrook Centre
Milton Road
Cambridge CB4 1YG, UK

P 01223 855340
W elifesciences.org
T @elife

eLife's transparent reporting form

We encourage authors to provide detailed information *within their submission* to facilitate the interpretation and replication of experiments. Authors can upload supporting documentation to indicate the use of appropriate reporting guidelines for health-related research (see EQUATOR Network), life science research (see the BioSharing Information Resource), or the ARRIVE guidelines for reporting work involving animal research. Where applicable, authors should refer to any relevant reporting standards documents in this form.

If you have any questions, please consult our Journal Policies and/or contact us: editorial@elifesciences.org.

Sample-size estimation

- You should state whether an appropriate sample size was computed when the study was being designed
- You should state the statistical method of sample size computation and any required assumptions
- If no explicit power analysis was used, you should describe how you decided what sample (replicate) size (number) to use

Please outline where this information can be found within the submission (e.g., sections or figure legends), or explain why this information doesn't apply to your submission:

No explicit power analysis was used, as we used datasets that were available and previously generated. For the population genetics analyses of long-range LD, our discovery cohort was a smaller sample size and we explicitly looked for a larger dataset to confirm the findings, with the largest Peruvian cohort available with genotype data used in this study.

Replicates

- You should report how often each experiment was performed
- You should include a definition of biological versus technical replication
- The data obtained should be provided and sufficient information should be provided to indicate the number of independent biological and/or technical replicates
- If you encountered any outliers, you should describe how these were handled
- Criteria for exclusion/inclusion of data should be clearly stated
- High-throughput sequence data should be uploaded before submission, with a private link for reviewers provided (these are available from both GEO and ArrayExpress)

Please outline where this information can be found within the submission (e.g., sections or figure legends), or explain why this information doesn't apply to your submission:

For RNA-seq, the CETP knocked down (KD) experiments in hepatocyte HepG2 cells were done using five biological replicates and five control replicates. We deposited the data on GEO (embargoed data set, NCBI Gene Expression Omnibus, accession number GSE174640). For qPCR of CETP-KD, it is five biological replicates and five control replicates, for ADCY9-KD and CETP/ADCY9 overexpression, it is four biological replicates and four control replicates for each. For western blot of CETP-KD, it is three biological replicates and three control replicates. Information about these are in Methods and Appendix.

eLife Sciences Publications, Ltd is a limited liability non-profit non-stock corporation incorporated in the State of Delaware, USA, with company number 5030732, and is registered in the UK with company number FC030576 and branch number BR015634 at the address Westbrook Centre, Milton Road, Cambridge CB4 1YG.

Statistical reporting

- Statistical analysis methods should be described and justified
- Raw data should be presented in figures whenever informative to do so (typically when N per group is less than 10)
- For each experiment, you should identify the statistical tests used, exact values of N, definitions of center, methods of multiple test correction, and dispersion and precision measures (e.g., mean, median, SD, SEM, confidence intervals; and, for the major substantive results, a measure of effect size (e.g., Pearson's r, Cohen's d)
- Report exact p-values wherever possible alongside the summary statistics and 95% confidence intervals. These should be reported for all key questions and not only when the p-value is less than 0.05.

Please outline where this information can be found within the submission (e.g., sections or figure legends), or explain why this information doesn't apply to your submission:

P-value are presented throughout the Results section, with the specific tests used outlined in each case. When permutation tests are used and the value derived from the data is outside the null distribution created by permutation, the p-value is reported as "smaller than" 1 divided by the number of random samples. Permutation tests are described in Methods and Appendix 1. P-values are also presented in figures, with figure legends detailing the tests used. All statistical tests are done with R or SAS and the functions used are reported in the Methods section and Appendix 1.

(For large datasets, or papers with a very large number of statistical tests, you may upload a single table file with tests, Ns, etc., with reference to sections in the manuscript.)

Group allocation

- Indicate how samples were allocated into experimental groups (in the case of clinical studies, please specify allocation to treatment method); if randomization was used, please also state if restricted randomization was applied
- Indicate if masking was used during group allocation, data collection and/or data analysis

Please outline where this information can be found within the submission (e.g., sections or figure legends), or explain why this information doesn't apply to your submission:

This information doesn't apply to our submission: we do not present data from an interventional study and there was no group allocation.

Additional data files ("source data")

- We encourage you to upload relevant additional data files, such as numerical data that are represented as a graph in a figure, or as a summary table
- Where provided, these should be in the most useful format, and they can be uploaded as "Source data" files linked to a main figure or table
- Include model definition files including the full list of parameters used
- Include code used for data analysis (e.g., R, MatLab)
- Avoid stating that data files are "available upon request"

Please indicate the figures or tables for which source data files have been provided:

We have included all source code and numerical data for generating the main figures and figures supplements of the paper. This information can be accessed through : https://github.com/HussinLab/adcy9_cetp_Gamache_2021

eLife Sciences Publications, Ltd is a limited liability non-profit non-stock corporation incorporated in the State of Delaware, USA, with company number 5030732, and is registered in the UK with company number FC030576 and branch number BR015634 at the address Westbrook Centre, Milton Road, Cambridge CB4 1YG.
